# A Novel Bispecific Antibody against Human CD3 and Ephrin Receptor A10 for Breast Cancer Therapy

**DOI:** 10.1371/journal.pone.0144712

**Published:** 2015-12-17

**Authors:** Shintaro Taki, Haruhiko Kamada, Masaki Inoue, Kazuya Nagano, Yohei Mukai, Kazuma Higashisaka, Yasuo Yoshioka, Yasuo Tsutsumi, Shin-ichi Tsunoda

**Affiliations:** 1 Laboratory of Biopharmaceutical Research, National Institutes of Biomedical Innovation, Health and Nutrition, 7-6-8 Saito-Asagi, Ibaraki, Osaka, Japan; 2 Graduate School of Pharmaceutical Sciences, Osaka University, 1–6 Yamadaoka, Suita, Osaka, Japan; 3 The Center for Advanced Medical Engineering and Informatics, Osaka University, 1–6 Yamadaoka, Suita, Osaka, Japan; 4 Center for Drug Design Research, National Institutes of Biomedical Innovation, Health and Nutrition, 7-6-8 Saito-Asagi, Ibaraki, Osaka, Japan; Mie University Graduate School of Medicine, JAPAN

## Abstract

Ephrin receptor A10 (EphA10), a transmembrane receptor that binds to ephrin, is a newly identified breast cancer marker protein that has also been detected in HER2-negative tissue. In this study, we report creation of a novel bispecific antibody (BsAb) binding both EphA10 and CD3, thereby forming a bridge between antigens expressed on both tumor and immune cells and promoting recognition of tumor cells by immune cells and redirection of cytotoxic T cells (CTL). This BsAb (EphA10/CD3) was expressed in supernatants of BsAb gene-transfected cells as monomeric and dimeric molecules. Redirected T-cell lysis was observed when monomeric and dimeric BsAb were added to EphA10-overexpressing tumor cells *in vitro*. Furthermore, dimeric BsAb (EphA10/CD3) was more cytotoxic than monomeric BsAb, with efficient tumor cell lysis elicited by lower concentrations (≤10^−1^ μg/mL) and a lower effector to target (E/T) cell ratio (E/T = 2.5). Dimeric BsAb (EphA10/CD3) also showed significant anti-tumor effects in human xenograft mouse models. Together, these results revealed opportunities to redirect the activity of CTL towards tumor cells that express EphA10 using the BsAb (EphA10/CD3), which could be tested in future clinical trials as a novel and potent therapeutic for breast cancer tumors.

## Introduction

Monoclonal antibodies (mAbs) are important therapeutic molecules in the treatment of hematologic malignancies as well as solid tumors [1–4]. Trastuzumab, a humanized mAb directed against human HER2, was approved for clinical use for patients with HER2-overexpressing metastatic breast cancer [5]. The anti-proliferative and cytotoxic effects of trastuzumab likely result from a combination of antibody-dependent cellular cytotoxicity (ADCC), inhibition of extracellular domain cleavage, decreased intracellular signal transduction and anti-angiogenic effects [6]. However, many breast cancer patients with low-level or no HER2 expression do not respond to trastuzumab therapy. Furthermore, the majority of trastuzumab-treated patients will develop resistance to trastuzumab within one year of treatment initiation [7–11]. Moreover, the FcγRIII 158V/F polymorphism interferes with the ability to generate ADCC responses [12, 13]. Therefore, there is a significant need for novel biomarkers and anti-tumorigenic therapeutics for HER2-negative breast cancer patients.

Ephrin receptors, which are a subfamily of receptor tyrosine kinases (RTKs), play important roles in cell–cell communications regulating cell attachment, shape, and mobility in neuronal and endothelial cells [14–16]. Members of the Ephrin receptor family induce the formation of neural networks and promote angiogenesis, and they are overexpressed in various cancer cells [17–19]. Previously, we identified Ephrin receptor A10 (EphA10) as a new human breast cancer biomarker by proteomic analysis [20]. In the Immunohistological (IHC) studies, 49% of breast cancer tissues (93/189) and 44% of HER2 negative tissues (60/136) were EphA10-positive while normal breast tissues were negative. Furthermore, EphA10 was not expressed in 36 normal tissues, with the exception of testis [21]. We also confirmed the testis-specific expression profile of EphA10 at the mRNA level via real-time quantitative PCR methods. Almost all breast cancer patients are female, and therefore EphA10 targeted therapy is a promising therapeutic strategy for most HER2-negative breast cancer patients.

Recently, immune cell-mediated antitumor therapy has been achieved more effectively using bispecific antibodies (BsAb) that bind to surface target antigens on both cancer cells and immune cell such as T-cells [22, 23], NK cells [24, 25] and macrophages [26, 27]. Most BsAb have targeted the CD3 complex on T cells because cytotoxic T cells are some of the most potent killer cells of the immune system. However, monoclonal full IgG therapy is not effective in directly engaging T cells because they lack Fc-gamma receptors. In particular, a bispecific T-cell engager (BiTE), which has the potential to redirect tumor resident and cytotoxic T cells (CTL), showed significant cytotoxicity against tumor cells [22, 23, 28–30]. Actually, the trifunctional Ertumaxomab (HER2/CD3) showed high efficacy for breast cancer patients who express low levels of HER2 and therefore do not respond well to trastuzumab [31]. The development of novel therapeutics is essential because current therapies are inadequate to treat most HER2-negative breast cancer patients.

In this study, we report the construction of a novel BsAb that targets EphA10, thus resembling BiTE technology. We also demonstrate the specificity and potent efficacy of redirected target cell lysis and antitumor activity of BsAb (EphA10/CD3) using both *in vitro* and *in vivo* methods. Our findings demonstrate that BsAb (EphA10/CD3) could potentially be used to achieve potent antitumor T-cell responses in EphA10-positive breast cancer patients.

## Materials and Methods

### Cell lines and culture

Expi293F cells (Invitrogen; Life Technologies; Carlsbad, CA) were cultured in shaker incubators (37°C, 8% CO_2_) in Expi293 Expression Medium. Hybridoma OKT3 (CRL-8001), MDA-MB-435 (human cancer cell line; HTB-129) and Jurkat (human T lymphocyte; TIB-152) cells were obtained from American Type Culture Collection (ATCC, Rockville, MD) and cultured under the recommended conditions. Human cells that overexpressed EphA10, MDA-MB-435 (MDA-MB-435^EphA10^), were established in our laboratory. In brief, a lentiviral vector encoding human EphA10 was transfected into MDA-MB-435 cells and stably transfected cells were obtained by Blasticidin (Invitrogen) selection. A hybridoma producing anti-EphA10 IgG was established from splenocytes of a human EphA10-immunized mouse by fusion with a mouse myeloma line. No authentication was done by the authors.

### Preparation of PBMC

PBMCs were prepared from the peripheral blood of healthy donors. All the healthy doners gave their written informed consent to participate in the study according to the Helsinki declaration. The study protocol was approved by the local ethics committee (Institutional Review Board of the National Institutes of Biomedical Innovation, Health and Nutrition registered under the number 78 detailed on its website. http://www.nibio.go.jp/part/strategy/ethics/pdf/rinrisinsa_31.pdf)

### Cloning of variable (V) immunoglobulin domains

The genes of V light-chain (VL) and V heavy-chain (VH) domains from each hybridoma were subcloned using 5'-Full RACE kits (Takara Bio, Kyoto, Japan). The amplified DNA was directionally subcloned into a plasmid vector using the TOPO TA cloning kit (Invitrogen) and sequenced using a 3130xl Genetic Analyzer (Applied Biosystems, Carlsbad, CA).

### Vector construction

The vectors to express the bispecific antibody or single chain Fv (scFv), respectively, were constructed as described previously [32]. The primer sequences are shown in Table 1.

**Table 1:** Oligonucleotide sequences of PCR primers used for construction of BsAb vectors.

Name of primer	Nucleotide sequence (5’-3’)*
5’*Nco*I-VL (hEphA10)	NNNCCATGGCCAGTTTTGTGATGACCCAGACTCCC
3’VL (hEphA10)-Linker	AGAGCCGCCGCCGCCGCTACCACCACCACCAGCCCGTTTGATTTCCAGCTTGG
5’Linker-VH (hEphA10)	GGCGGCGGCGGCTCTGGTGGTGGTGGATCCCAGGTTCTGCTGCAGCAGTCT
3’VH (hEphA10)-*Not*I	NNNGCGGCCGCTGAGGAGACGGTGACTGAGGTT
5’*Nco*I-VL (hCD3)	NNNCCATGGCCCAAATTGTTCTCACCCAGTCTCCAG
3’VL (hCD3)-Linker	AGAGCCGCCGCCGCCGCTACCACCACCACCTTTCAGCTCCAGCTTGGTCCC
5’Linker-VH (hCD3))	GGCGGCGGCGGCTCTGGTGGTGGTGGATCCCAGGTCCAGCTGCAGCAGT
3’VH (hCD3)-*Not*I	NNNGCGGCCGCTGAGGAGACTGTGAGAGTGGTG
5’*Hin*dIII-SP-VL(hCD3)	NNNAAGCTTGTCGACCATGGGATGGTCACTGATTCTGCTGTTTCTGGTCGCCGTCGCTACTGGTGTCCACTCAGATATCCAAATTGTTCTCACCCAGTCTC
3’VH (hCD3)-Linker’	ACTGCTACCACCACCACCTGAGGAGACTGTGAGAGTGGTG
5’Linker’-VL(hEphA10)	TCAGGTGGTGGTGGTAGCAGTTTTGTGATGACCCAGACTCCC
3’VH (hEphA10)-His tag-*Not*I	NNNGCGGCCGCTCAATGATGATGATGATGATGTGAGGAGACGGTGACTGAGGT
5’Linker’-VL(His)	TCAGGTGGTGGTGGTAGCAGTTTTGTGATGACCCAGACTCCC
3’VH (His)-His tag-*Not*I	NNNGCGGCCGCTCAATGATGATGATGATGATGTGAGGAGACGGTGACTGAGGT

*The restriction enzyme site is underlined.

For subcloning of target genes, the *E*. *coli* TOP10 strain (Invitrogen) was used. To obtain an anti-EphA10 scFv and an anti-CD3 scFv fragment, the corresponding VL and VH regions were cloned into separate vectors as templates for VL- and VH-specific PCR using the primer pairs 5’ *Nco*I-VL (hEphA10 or hCD3) / 3’ VL (hEphA10 or hCD3)-Linker and 5’ Linker-VH (hEphA10 and hCD3) / 3’ VH (hEphA10 or hCD3)-*Not*I, respectively. Overlapping complementary sequences were introduced into the PCR products that combined to form the coding sequence of the 15-amino acid (G_4_S)_3_ Linker during the subsequent fusion PCR. This amplification step was performed with the primer pair 5’ *Nco*I-VL (hEphA10 or hCD3) / 3’ VH (hEphA10 or hCD3)-*Not*I, and the resulting fusion product was cleaved with the restriction enzymes *Nco*I and *Not*I, then cloned into the pET20b vector (Invitrogen). Next, to construct the bispecific antibody (EphA10/CD3) expression vector, two previously described scFv vectors were used as templates for scFv-specific PCR with the primer pairs 5’ *Hin*dIII-Signal Peptide (SP; MGWSLILLFLVAVATGVHS)-VL (hCD3) / 3’ VH (hCD3)-Linker’ and 5’ Linker’-VL (hEphA10) / 3’ VH (hEphA10)-His tag -*Not*I, respectively. Overlapping complementary sequences were introduced into the PCR products that combined to form the coding sequence of the 5-amino acid G_4_S Linker during the subsequent fusion PCR. This amplification step was performed with the primer pair 5’ *Hin*dIII-SP-VL (hCD3) / 3’ VH (hEphA10)-His tag-*Not*I, and the resulting fusion product was cleaved with the restriction enzymes *Hin*dIII and *Not*I, then cloned into the pcDNA3.1 (+) vector (Invitrogen). The amino acid sequence of BsAb (EphA10/CD3) is shown in S1 Fig. Furthermore, a vector for the control bispecific antibody (His/CD3) was prepared from an anti- hexahistidine tag (His tag) scFv and an anti-CD3 scFv fragment, following the same procedure.

### Expression and purification of BsAb (EphA10/CD3)

BsAb (EphA10/CD3) and BsAb (His/CD3) were prepared using the Expi293 Expression System (Invitrogen). Plasmid DNA (300 μg) and ExpiFectamin 293 Reagent (800 μL) were mixed with Opti-MEM® I medium (15 mL final volume) and allowed to stand at room temperature for 25 min. The mixed solution was added to 7.5 x 10^8^ Expi293 cells cultured in Expi293 Expression Medium and gently mixed in a shaker incubator at 37°C with a humidified atmosphere of 8% CO2 in air. At 18 hours post-transfection, 1.5 mL of ExpiFectamin 293 Transfection Enhancer 1 and 15 mL of ExpiFectamin 293 Transfection Enhancer 2 were added to each flask. The transfected cells were then incubated under the same conditions in a shaking incubator for one week.

Each BsAb was purified from the cell culture supernatant by immobilized metal affinity chromatography (IMAC) and gel filtration chromatography with a Superdex200 prep grade column (GE Healthcare, Little Chalfont Bucks, UK) equilibrated in phosphate-buffered saline (PBS). SDS-PAGE and western blot analysis were performed to detect and confirm the size and purity of each BsAb. Purified proteins were concentrated in PBS by ultrafiltration using a Centriprep^®^ 30K or 50K device (Millipore, Billerica, MA, USA), and protein concentrations were estimated using a Coomassie Plus Protein Assay (Thermo Fisher Scientific, Rockford, IL).

### Flow cytometric analysis

5 x 10^5^ cells (MDA-MB-435, MDA-MB-435^EphA10^, Jurkat) were used for flow cytometry. Each cell was suspended in Suspension buffer (2% FBS containing PBS) and incubated with 6 μg monomeric or dimeric BsAb or control IgG (anti-EphA10, anti-CD3) for 1 hour on ice, respectively. After washing with Suspension buffer, the cells were incubated with Surelight P3 labeled antibodies against the His tag (Columbia Biosciences, Frederick, MD) and Surelight P3 labeled antibodies against the mouse IgG (Columbia Biosciences) for 1 hour on ice. The cells were washed again and resuspended in 500 μL Suspension buffer and flow cytometric analysis was performed (FACScanto; BD Biosciences, San Jose, CA). All tests were carried out in triplicate.

### Cytotoxicity assays

Cytotoxicity assays were performed as described previously with slight modifications [32]. In brief, human PBMCs as effector cells were isolated from healthy donors. MDA-MB-435^EphA10^ cells and MDA-MB-435 parent cells were used as target cells. Target cells (10^3^ cells/well) were added to 96-well plates (NUNC; Life Technologies, Gaithersburg, MD) with 10% FBS containing D-MEM (Wako, Osaka, Japan) at 37°C in a humidified atmosphere containing 5% CO_2_. After over-night culture, supernatants were removed and non-stimulated PBMC were added to an effector-to-target (E/T) ratio of 2.5 or 5 with each of the antibodies (10^−2^–10 μg/mL), respectively. In this case, Anti-EphA10 IgG and anti-CD3 IgG were prepared from Hybridomas. After 48 hours of incubation, lactate dehydrogenase (LDH) released into the supernatant was measured using a CytoTox 96® non-radioactive cytotoxicity assay (Promega, Madison, WI). Percentages of specific lysis were calculated according to the formula: % cytotoxicity = [(experimental release)—(effector spontaneous release)—(target spontaneous release)] / [(target maximum release)—(target spontaneous release)] x 100. All tests were carried out in triplicate.

### Analysis of Th1 cytokine production

Two plates containing MDA-MB-435^EphA10^ cancer cells (10^3^ cells/well) or no target cells, respectively, were cultured as described. After overnight culture, non-stimulated PBMC (5 x 10^3^ cells/well) were added to each plate and incubated with various concentrations of each BsAb and control full IgG. Th1 Cytokine (IFN-γ, IL-2 and TNF-α) concentrations in the supernatants were analyzed using an Opti-ELISA kit (BD Biosciences) following the manufacturer’s instructions. All tests were carried out twice.

### *In vivo* efficacy of BsAb (EphA10/CD3) against a xenograft model

*In vivo* efficacy of BsAb (EphA10/CD3) was evaluated using a xenograft model that consisted BALB/c nu/nu mice (Japan SLC, Inc., Shizuoka, Japan) that received a s.c. engraftment of 1 x 10^6^ MDA-MB-435^EphA10^ cells with 1 x 10^6^ non-stimulated PBMC. Six animals per group were treated intravenously with 1 or 10 μg dimeric BsAb (EphA10/CD3), 10 μg dimeric BsAb (His/CD3) and 10 μg control full IgG (anti-EphA10, anti-CD3) administered on study days 0, 1, 2 and 3. Tumor growth in two perpendicular directions was measured on the indicated days with calipers and tumor volumes (mm^3^) were calculated using the formula: V = (width^2^ x length) / 2. All experimental procedures were conducted in accordance with the Japanese regulations on animal experiments and approved by the Institutional Animal Care and Use Committee of National Institutes of Biomedical Innovation, Health and Nutrition, Osaka, Japan.

## Results

### Formulation of BsAb (EphA10/CD3)

**Fig 1:**
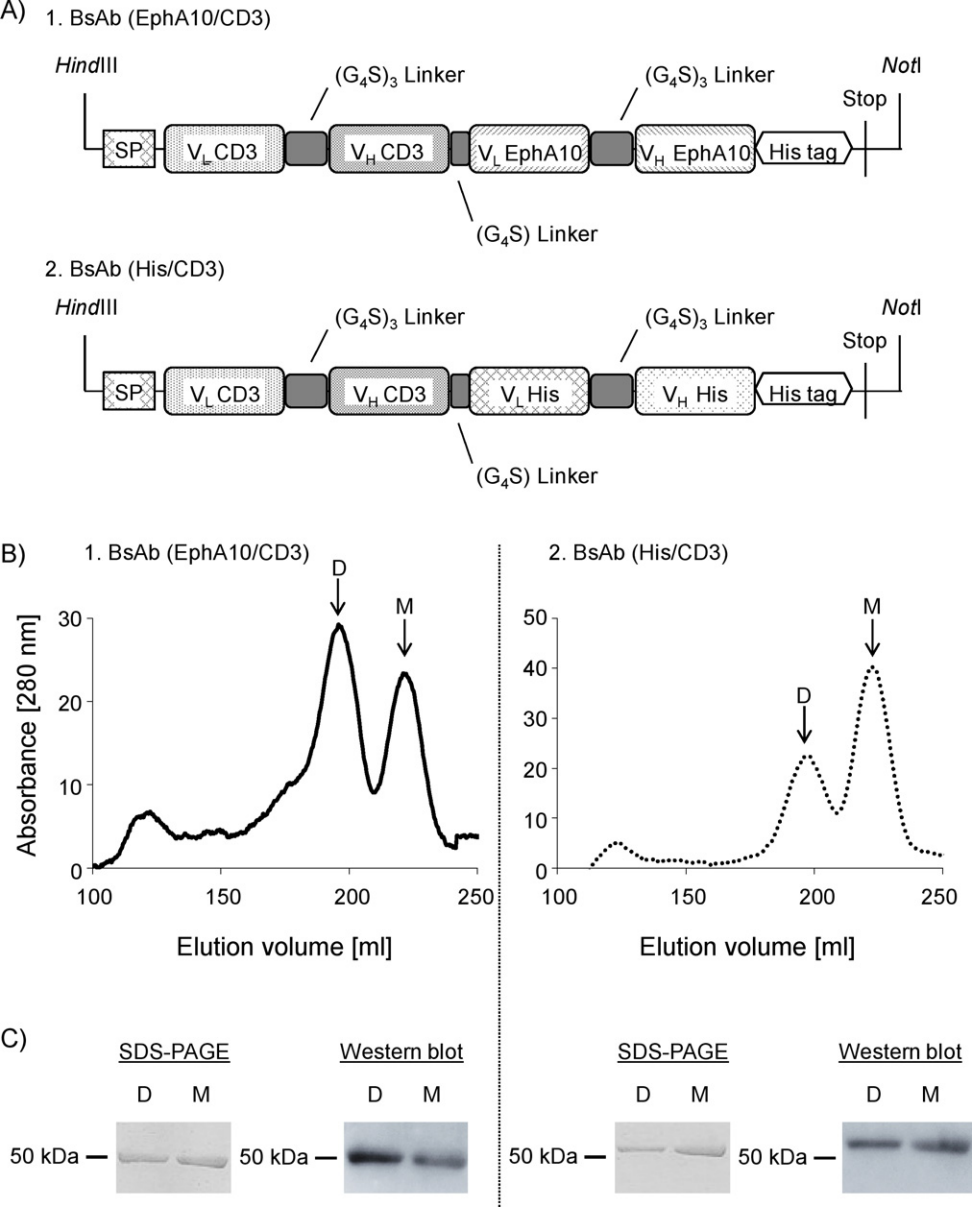
Characteristics of BsAb (EphA10/CD3) and BsAb (His/CD3). (A) Gene construct of operons encoding each BsAb in plasmid pcDNA3.1. (B) Gel filtration chromatography profile of BsAb (EphA10/CD3) and BsAb (His/CD3), which were purified with IMAC. (C) SDS-PAGE and Western blot analysis of dimer (lane D) and monomer (lane M) fractions. The parallel line shows molecular size standards with their apparent molecular weights in kiloDalton (kDa). The down arrow shows elution peak of monomeric and dimeric BsAb. Abbreviations: D is dimer, M is monomer.

Each BsAb was constructed with the single-chain Fv fragment (V_L_-(G_4_S)_3_-V_H_) derived from the hybridomas (anti-EphA10 IgG, anti-CD3 IgG) or phage library (anti-His scFv) and then connected by a G_4_S linker (Fig 1A). The plasmid vector construct was designed by adding an N-terminal signal peptide to express BsAb in a soluble form and adding a C-terminal hexahistidine tag (His tag) to purify it using affinity chromatography on a Ni-Sepharose column. This plasmid vector was transfected into Expi293 cells. Western blot analysis of a small-scale culture (30 mL) revealed that each BsAb was expressed in culture supernatants (data not shown), thus large-scale culture (300 mL) was performed. Pooled supernatants were purified by IMAC, and eluted fractions containing each BsAb were further purified by gel-filtration chromatography, which produced two main peaks, respectively (Fig 1B). SDS-PAGE under reducing conditions followed by western blot analysis showed only a single band indicating a ~50 kDa protein (Fig 1C), consistent with the calculated molecular mass of approximately 53 kDa for each BsAb. Calculated molecular weights of prepared samples, determined using a calibration curve from samples with defined molecular weights run over a gel-filtration chromatography column, are shown in S1 Table. These results suggested that the BsAb (EphA10/CD3) and the BsAb (His/CD3) existed in both monomeric and dimeric forms in the cell supernatants. Other groups have also reported that BsAb of BiTE class could be produced as both monomers and dimers [33, 34]. We expected that the characteristics of the BsAb dimer would differ from those of the monomer. Therefore, the binding specificity and cytotoxic activity of each BsAb species was evaluated.

### Binding specificity of BsAb (EphA10/CD3) for human EphA10 and CD3

**Fig 2:**
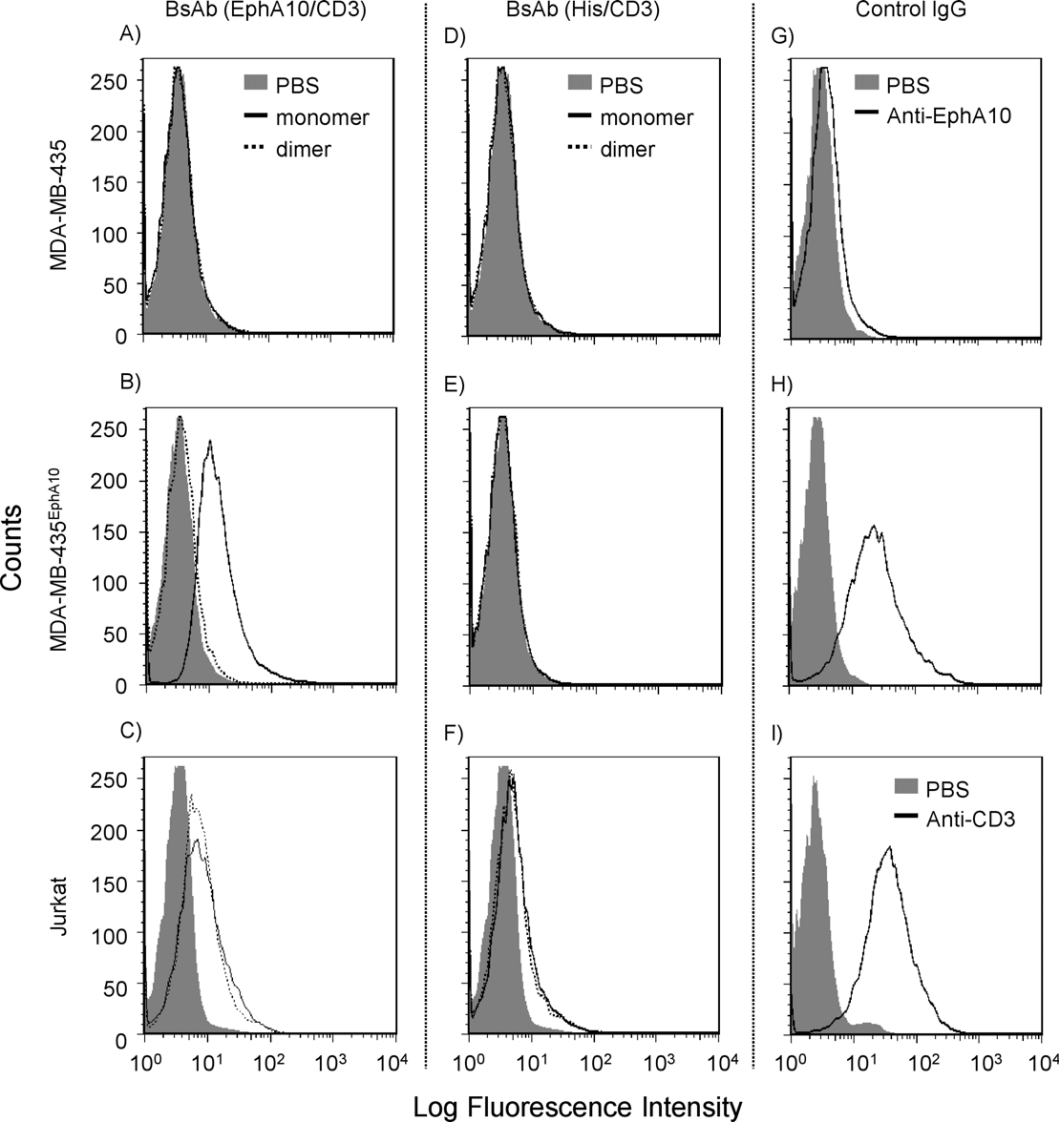
Binding activity of monomeric and dimeric BsAbs. The left panels (A, B, C) are monomeric and dimeric BsAbs (EphA10/CD3) and the central panels (D, E, F) are monomeric and dimeric BsAbs (His/CD3) and the right panels (G, H, I) are control full IgG (anti-EphA10, anti-CD3). MDA-MB-435 parental cells, human EphA10 transfected cells (MDA-MB-435^EphA10^) and Jurkat cells (CD3 positive) were used for flow cytometric analysis. Binding activities of each antibody sample were measured at 6 μg. Cell-binding proteins were detected via SureLight P3 conjugated anti-His tag or anti-mouse IgG mAb. Gray filled-in area is vehicle control (PBS). Line indicates each antibody sample (dotted lines are dimeric BsAb).

Binding activities of monomeric and dimeric BsAb (EphA10/CD3) were examined by flow cytometric analysis using the MDA-MB-435 parental cells, MDA-MB-435^EphA10^ cells and Jurkat cells. Specific binding of both EphA10 and CD3 antigens was observed for monomeric and dimeric BsAb (EphA10/CD3) (Fig 2). In this case, Dimeric BsAb (EphA10/CD3) could not be detected, however, we could confirm its binding activity via surface plasmon resonance (SPR) (S2 Fig). On the other hand, monomeric and dimeric BsAb (His/CD3) could bind CD3 not but EphA10. These results demonstrated that the binding activity of each domain was preserved after the bispecific format conversion. Furthermore, the structural differences between monomeric and dimeric forms did not significantly affect the binding activity.

### Redirected target cell lysis of BsAb (EphA10/CD3) with PBMC

**Fig 3:**
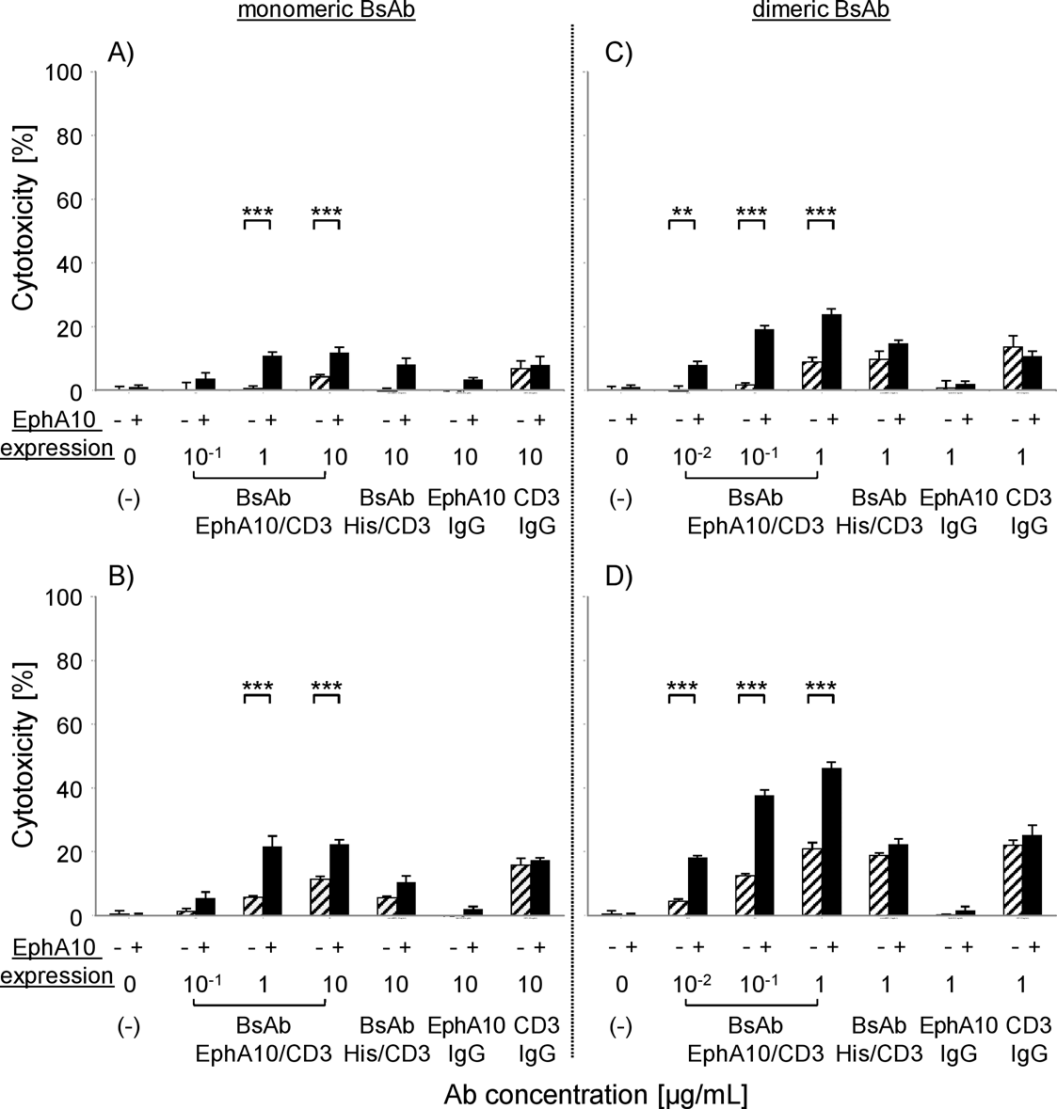
*In vitro* cytotoxicity of BsAb (EphA10/CD3) against MDA-MB-435^EphA10^ and parental cells. The left panels are monomeric BsAb (A, B) and the right panels are dimeric BsAb (C, D). MDA-MB-435 parental cells (slashed column) and MDA-MB-435^EphA10^ (black column) cells were co-cultured with human PBMC at E/T ratios of 2.5 (A, C) and 5 (B, D). Each point represents the mean of triplicate determinations; Error bars represent the standard deviations of triplicate determinations. Asterisks label readings that were statistically significant (unpaired Student’s T-test) from MDA-MB-435 and MDA-MB-435^EphA10^ (***: P<0.001, **: P<0.01).

The efficacy of T-cell mediated redirected lysis of MDA-MB-435^EphA10^ cells and the parental cells following administration of BsAb (EphA10/CD3) was examined using an LDH cytotoxicity assay. Non-stimulated PBMC were used as effector cells at E/T ratios of 2.5 and 5, respectively. As shown in Fig 3, monomeric and dimeric BsAb (EphA10/CD3) showed potent, significant target-specific cytotoxicity against MDA-MB-435^EphA10^ cells compared with the full IgG constructs (anti-EphA10 IgG, anti-CD3 IgG) and BsAb (His/CD3) in a dose-dependent manner. Furthermore, the cytotoxic efficacy of dimeric BsAb (EphA10/CD3) was higher than that of the monomeric form at low antibody concentrations and low E:T ratios, indicating that dimerization would improve the avidity of binding to each antigen. We also confirmed the target-specific cytotoxicity of BsAb (EphA10/CD3) by testing other EphA10-positive cancer cell lines (S3 and S4 Figs). Although anti-CD3 IgG and dimeric BsAb (His/CD3) showed similar cytotoxicity against both types of cells, this might have been caused by non-specific activation of the PBMC. In this study, we tried to determine the EC50 values of all antibody samples, but we could not get credible data because of the small concentration range (only three different points could be tested). However, dimeric BsAb (EphA10/CD3) showed a significant cytotoxic effect against MDA-MB435^EphA10^ compared with control samples.

### Th1 cytokine production from PBMC by BsAb (EphA10/CD3) stimulation

**Fig 4:**
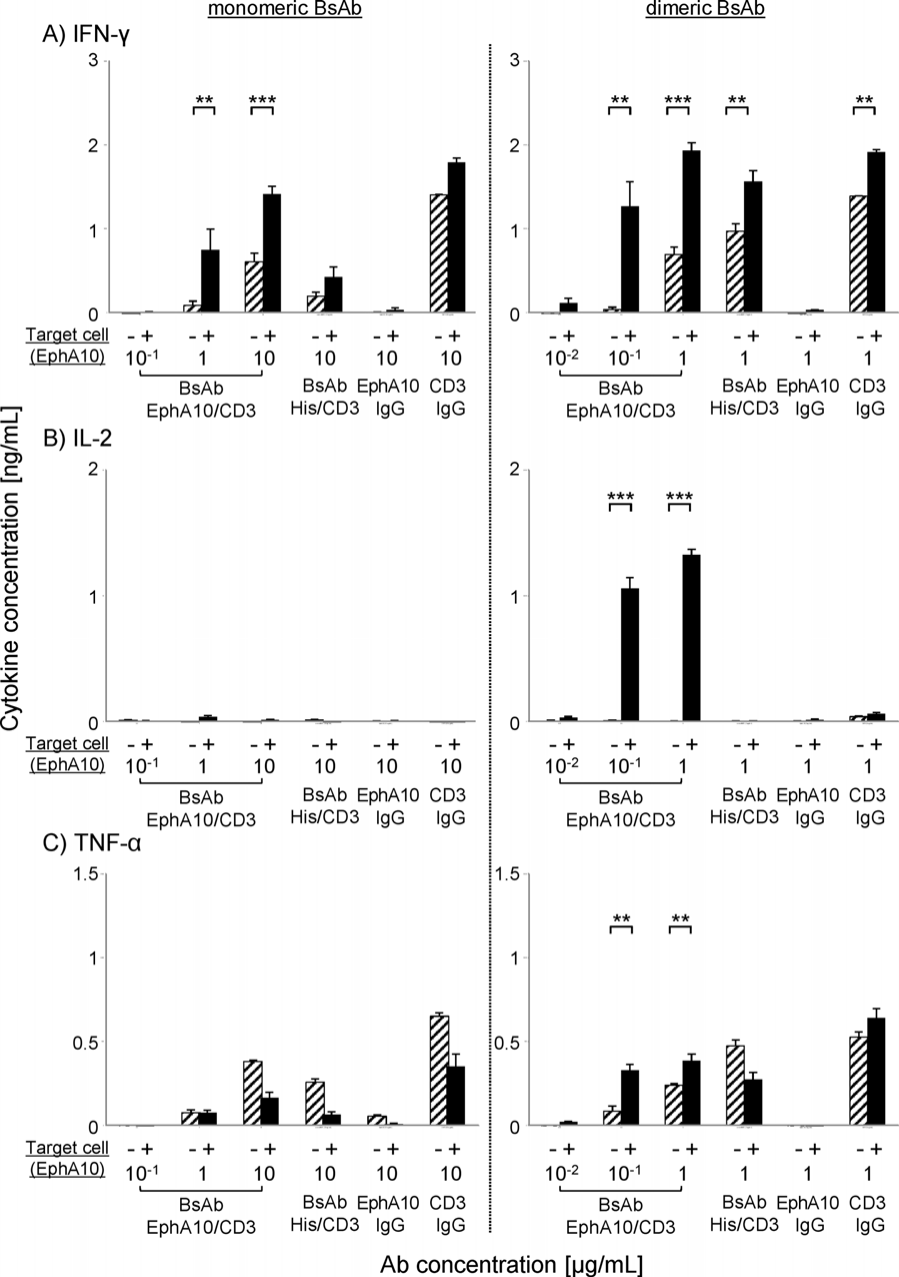
Th1 cytokine production from human PBMCs after BsAb (EphA10/CD3) reaction. Non-stimulated PBMC (E/T ratio of 5) were incubated in the presence of MDA-MB-435^EphA10^ breast cancer cells (black column) or no target cells (slashed column) with various concentrations of each BsAb (EphA10/CD3) and control sample (BsAb (His/CD3), full IgG). The production of (A) IFN-γ, (B) IL-2 and (C) TNF-α in the presence of monomer (left panel; 1–10 μg/mL) or dimer (right panel; 0.1–1 μg/mL) was determined from the cell culture supernatants. Control samples were incubated with BsAb (His/CD3) (1 or 10 μg/mL) or anti-EphA10 IgG (1 or 10 μg/mL) or anti-CD3 IgG (1 or 10 μg/mL). Each point represents the mean of triplicate determinations; Error bars represent the standard deviations of triplicate determinations. Asterisks label readings that were statistically significant (unpaired Student's T-test) from PBMC with or without MDA-MB-435^EphA10^ (***: P<0.001, **: P<0.01).

Other groups have reported that Th1 cytokines were secreted from PBMCs in the presence of target cells together with BsAb [33], thus Th1 cytokines were measured to verify the event of T-cell redirected tumor lysis. The immune stimulation of effector cells against target cells by BsAb (EphA10/CD3) was evaluated by measurement of IFN-γ, IL-2 and TNF-α production from activated PBMCs. The results showed that BsAb (EphA10/CD3) induced the production of IFN-γ, IL-2 and TNF-α from activated PBMC (Fig 4). Furthermore, the production of cytokines from PBMC was enhanced in the presence of target cells but not without these cells. This result was consistent with cytotoxicity assay results (Fig 3), and it suggested that the activation of T cells is effectively enhanced only when BsAb (EphA10/CD3) interact with cells expressing both of the target antigens. The production of IL-2 was accelerated in the presence of target cells and effector cells only with the dimeric form of the BsAb (EphA10/CD3), although anti-CD3 IgG and control BsAb (His/CD3) showed similar activation of PBMC not dependent on target cells. These results support our conclusion that the dimeric BsAb (EphA10/CD3) was more cytotoxic than the monomeric BsAb (EphA10/CD3) as a control Ab at low concentrations and low E/T ratios. Because IL-2 has the ability to potently stimulate CTL and NK cells, the dimeric BsAb is an attractive candidate for cancer immunotherapy [35].

### *In vivo* efficacy of BsAb (EphA10/CD3) in xenograft model

**Fig 5:**
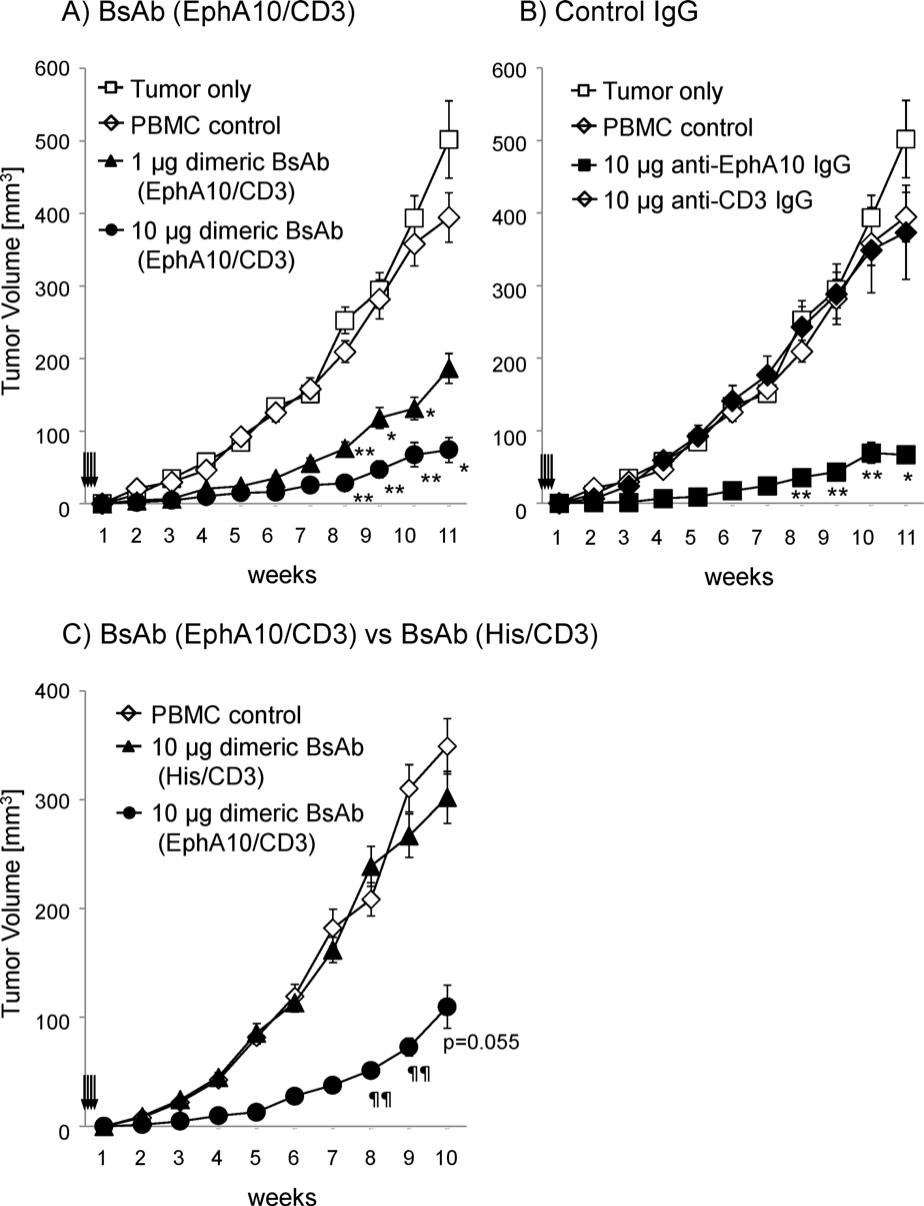
Dose-dependent effect of dimeric BsAb (EphA10/CD3) in BALB/c nu/nu mice. Each mouse (n = 6) was inoculated subcutaneously with a mixture of 10^6^ MDA-MB-435^EphA10^ cells and 10^6^ human PBMC at an E/T ratio of 1 and the indicated doses of dimeric BsAb (EphA10/CD3) were administered intravenously on study days 0 to 3 (arrows). A) Mean values of tumor growth curves are shown for mice that were untreated (⬜) or only PBMC-treated (◇), or treated with PBMC and 1 μg dimeric BsAb (EphA10/CD3) (▲) or 10 μg dimeric BsAb (EphA10/CD3) (●). B) Mean values of tumor growth curves are shown for mice that were treated with PBMC and 10 μg anti-EphA10 IgG (■) or 10 μg anti-CD3 IgG (◆). C) Mean values of tumor growth curves are shown for mice that were only PBMC-treated (◇), or treated with PBMC and 10 μg dimeric BsAb (EphA10/CD3) (●) or 10 μg dimeric BsAb (HIs/CD3) (▲). Values represent mean tumor sizes (in mm^3^) ± SEM (n = 6 per group). Asterisks label readings that were statistically significant (unpaired Student’s T-test) from the Ab treated group and the untreated group (**: P<0.01, *: P<0.05). Daggers indicate that differences were statistically significant from BsAb (EphA10/CD3) and BsAb (His/CD3) (¶¶: P<0.01).

Finally, the antitumor activity of dimeric BsAb (EphA10/CD3) was evaluated *in vivo*. The xenograft model mice inoculated with MDA-MB-435^EphA10^ cells were tested. In this experiment, 1 x 10^6^ non-stimulated human PBMC were injected with 1 x 10^6^ tumor cells into BALB/c nu/nu mice. At the beginning of the experiment, mice were then given daily i.v. doses of dimeric BsAb (EphA10/CD3) (1 or 10 μg/mouse) or dimeric BsAb (EphA10/CD3) (10 μg/mouse) or control full IgG (anti-EphA10, anti-CD3; 10 μg/mouse). The *in vivo* growth of MDA-MB-435^EphA10^ cells was the same as that of the parental cells in the presence or absence of PBMC. On the other hand, the groups treated with dimeric BsAb (EphA10/CD3) showed tumor growth that was significantly inhibited in a dose-dependent manner (Fig 5A). The antibody concentration range was almost the same as that reported for other BsAbs of the BiTE class [22, 29]. Furthermore, injection with 10 μg dimeric BsAb (EphA10/CD3) produced tumor inhibition just as potent as that of anti-EphA10 IgG (Fig 5B) and dimeric BsAb (His/CD3) had no effect (Fig 5C). S5 Fig shows our evaluation of the anti-tumor effect of another dimeric BsAb (EphA10/CD3’) that was constructed with anti-CD3 IgM. Dimeric BsAb (EphA10/CD3’) did not show an anti-tumor effect because its affinity for CD3 was very low (The dissociation constant, K_D_≒10^−7^). Thus, the dimeric BsAb (EphA10/CD3) showed effectiveness against EphA10-positive cancer tumors *in vivo* only when bound to both EphA10 and CD3.

## Discussion

Breast cancer patients are divided into the following four therapeutically-relevant subtypes on the basis of HER2, estrogen receptor (ER) and progesterone receptor (PR) expression levels in tumor cells; luminal A (HER2-, ER+ and/or PR+), luminal B (HER2+, ER+ and/or PR+), HER2-enriched (HER2+, ER-, PR-) and TNBC (HER2-, ER-, PR-). Currently available anticancer mAbs (e.g. Trastuzumab) recognize extracellular or cell surface proteins such as HER2, however, these proteins comprise only a small fraction of cellular proteins and are not completely tumor-specific. EphA10, originally identified as a breast cancer biomarker, is a cell surface receptor and a specific antigen for cancer tissue, including HER2-negative cases [20]. Furthermore, IHC studies revealed that the above four tumors subtypes contained the following percentages of EphA10-positive cells: luminal A (54%), luminal B (68%), HER2-enrich (64%), TNBC (67%). [21]. TNBC is an aggressive disease that is associated with a high proliferation index, visceral and central nervous system metastases, and poor outcomes [36–38]. Therefore, the identification of novel biological markers and generation of more efficient therapeutics against TNBC are needed. In this study, we examined the antitumor effect of BsAb (EphA10/CD3) by cytotoxic assays using PBMC and by administration of this BsAb to xenograft model mice. The results demonstrated a proof of this concept and indicated that EphA10-targeted therapy could be a highly potent new therapy against not only HER2-negative breast cancer but also against TNBC.

Similar to BsAb of the BiTE class, BsAb (EphA10/CD3) induced efficient tumor cell lysis at much lower concentrations than those required for standard mAb therapies. For example, the FDA approved Blinatumomab (human CD19/human CD3), which is a BiTE molecule against acute lymphoblastic leukemia (ALL). Blinatumomab showed highly potent efficacy at a low dose, which was approximately 1000-fold lower than the existing mAb drug doses used for Ph-relapsed/refractory B-precursor patients [39, 40]. The results of an *in vitro* cytotoxicity assay also demonstrated that a dimeric BsAb (EphA10/CD3) could induce redirected tumor killing by CTL at a low concentration (10^−1^ μg/mL). Furthermore, the *in vivo* efficacy of dimeric BsAb (EphA10/CD3) in a xenograft model showed that it could inhibit tumor growth at low doses (1, 10 μg), similar to the effective concentration ranges for other BsAbs [22, 29]. These results indicate that the dimeric BsAb is a potent new BiTE format. Secreted IL-2 is known to be a key factor for T-cell activation and for the anti-tumor effect of BsAb therapy [35]. Dimeric BsAb showed a more highly cytotoxic effect than monomeric BsAb because the production of IL-2 was accelerated only following administration of the dimeric form, as shown in Fig 4B. Multimerization of antibody fragments could improve not only the pharmacokinetic properties but also the rate and/or extent of tumor accumulation [41, 42]. For example, a single-chain diabody format molecule, Tandab, is a dimeric molecule that is expected to show improved potency and efficacy for the reasons described above, and it is now being tested in clinical trials [41, 43]. Thus, dimeric BsAb molecules appear to have significant advantages over monomeric BsAbs.

Although the dimeric form of the BsAb against EphA10/CD3 shows promise as a new therapeutic molecule for the treatment of EphA10-positive cancer, there are still potential problems that could affect its translation to the clinic. First, the drug design of BsAb (EphA10/CD3) should be optimized to improve its therapeutic effects. *In vivo* studies have indicated the dimeric BsAb (EphA10/CD3) is effective against EphA10-positive cancer tumors, however, we did not demonstrate a significant difference compared with anti-EphA10 IgG. In a previous report, we showed that anti-EphA10 IgG could induce CDC activity [44]. The anti-tumor effect of anti-EphA10 IgG *in vivo* was caused primarily by CDC effects. The present model could not be simply compared to the individual effects of BsAb and CDC. Furthermore, there is a possibility that this drug could elicit Human Anti-Mouse Antibodies (HAMA) [45] because the product was derived from mouse antibodies. Most antibody drugs are derived from humanized or human antibodies, which have a lower immunogenic potential [46]. In the future, the efficacy of humanized dimeric BsAb (EphA10/CD3) and humanized anti-EphA10 IgG should be compared in human clinical trials. We are currently developing human antibodies against EphA10 and CD3 antigens by using a human antibody library generated in our laboratory. Thus, we are now constructing various BsAbs (EphA10/CD3) from other BsAb formats (include tandem scFv and single-chain Diabody) and different anti-CD3 Ab in order to improve its anti-tumor effect. Of course, we should compare their characteristics *in vitro* and *in vivo*, including comparisons with monomeric constructs, to optimize the potential therapeutic effects. Next, it will be necessary to demonstrate the effect of BsAb (EphA10/CD3) against HER2-negative breast cancer derived from patients. The present study demonstrates a high efficacy of BsAb (EphA10/CD3) only against a cell line that overexpresses EphA10 in an early-stage xenograft model mouse. However, it is unclear whether this BsAb (EphA10/CD3) would be effective in terminal cancer patients. Thus, evaluation of other mouse models, for example, tumor-established models and TNBC tissue engrafted xenograft models, should be carried out in the future. The method of creating cancer tissue originated spheroid cells (CTOS) is the most powerful technology to evaluate such a mouse model [47]. This method could be used to generate highly purified and viable primary cancer cells from patients, which could be effectively prepared and cultured *in vitro*. Furthermore, CTOS formed xenograft tumors *in vivo* that retained the features of the parental tumors. Thus, it should be possible to evaluate the effect of BsAb (EphA10/CD3) both *in vitro* and *in vivo*, and results of these studies could predict clinical responses of breast cancer patients who are presently considered incurable.

In this study, we report the creation and initial testing of a novel BiTE class BsAb, (EphA10/CD3), which stimulates T cells to kill tumor cells that express EphA10 antigens. BsAb (EphA10/CD3) has two advantages as a potential novel breast cancer therapy. One is its specificity for a cancer-associated target molecule, and the other is the highly cytotoxic effect of the dimeric form of this BsAb against cancer cells. Our present findings suggest opportunities for using a BsAb (EphA10/CD3) to treat breast cancer patients whose tumors express EphA10. In the future, this type of BsAb could also be used as a novel drug to treat currently incurable cancers such as TNBC.

## Supporting Information

S1 Fig Amino acid sequence of BsAb (EphA10/CD3). (TIF)

S2 Fig Binding curves of monomeric and dimeric BsAbs (EphA10/CD3, His/CD3) against hEphA10 recombinant protein. Binding curves were determined by surface plasmon resonance (SPR) measurements with a BiacoreT200 (GE Healthcare). Anti-human antibodies (Human Antibody Capture Kit; GE Healthcare) were immobilized on CM5 chips (at ~10,000 RU) using standard amine-coupling chemistry. After hEphA10-Fc (5 μg/mL) was captured on anti-human antibodies, each BsAb sample (1–240 nM) was injected into the flow cell. Binding response was corrected by subtracting RU from a blank flow cell. A) monomeric BsAb (EphA10/CD3), B) monomeric BsAb (His/CD3), C) dimeric BsAb (EphA10/CD3), D) dimeric BsAb (His/CD3).
(TIF)

S3 Fig Flow cytometric analysis of anti-EphA10 IgG to MDA-MB-435EphA10, MDA-MB-468 (breast cancer cell line) and LN-Cap (prostate cancer cell line). The method was describedd in the manuscript.
(TIF)

S4 Fig *In vitro* cytotoxicity of BsAb (EphA10/CD3) against MDA-MB-468 and LN-Cap cells. The left panels are monomeric BsAb (A, B) and the right panels are dimeric BsAb (C, D). Upper panels are MDA-MB-468 and lower panels are LN-Cap. Target cells were co-cultured with human PBMC at E/T ratios of 5. Each point represents the mean of triplicate determinations; Error bars represent the standard deviations of triplicate determinations. Asterisks label readings that were statistically significant (unpaired Student’s T-test) from BsAb (EphA10/CD3) and BsAb (His/CD3) (**: P<0.01, *: P<0.05).
(TIF)

S5 Fig Dose-dependent effect of dimeric BsAb (EphA10/CD3) and dimeric BsAb (EphA10/CD3’) in BALB/c nu/nu mice. We evaluated the anti-tumor effect of another dimeric BsAb (EphA10/CD3’) that was constructed with anti-CD3 IgM.　Each mouse (n = 6) was inoculated subcutaneously with a mixture of 10^6^ MDA-MB-435^EphA10^ cells and 10^6^ human PBMC at an E/T ratio of 1 and the indicated doses of dimeric BsAb were administered intravenously on study days 0 to 3 (arrows). Mean values of tumor growth curves are shown for mice that were untreated (⬜) or only PBMC-treated (◇), or treated with PBMC and 10 μg dimeric BsAb (EphA10/CD3) (●),10 μg dimeric BsAb (EphA10/CD3’) (×). Values represent mean tumor sizes (in mm^3^) ± SEM (n = 6 per group). Section signs indicate statistically significant differences from BsAb (EphA10/CD3) and BsAb (EphA10/CD3’) (§§: P<0.01, §: P<0.05).
(TIF)

S1 Table Calculated molecular weights of each antibody via calibration curve of a gel-filtration chromatography column. Calibration curve was obtained by Gel Filtration Calibration Kit LMW and HMW (GE Healthcare).
(TIF)

## Abbreviations

EphA10: Ephrin receptor A10
BsAb: Bispecific antibody
mAbs: Monoclonal antibodies
ADCC: Antibody-dependent cellular cytotoxicity
RTKs: Receptor tyrosine kinases
BiTE: Bispecific T-cell engager
CTL: Cytotoxic T cells
ATCC: American Type Culture Collection
scFv: Single chain Fv
His: tag Hexahistidine tag
IMAC: Immobilized metal affinity chromatography
PBS: Phosphate-buffered saline
LDH: Lactate dehydrogenase
TNBC: Triple-Negative Breast Cancer
ALL: Acute lymphoblastic leukemia
HAMA: Human Anti-Mouse Antibodies
CTOS: Cancer tissue originated spheroid cells
