# Supplementary figures and images for: A Novel Bispecific Antibody against Human CD3 and Ephrin Receptor A10 for Breast Cancer Therapy
Source: PLoS One. 2015 Dec 17;10(12):e0144712. doi: 10.1371/journal.pone.0144712 (PMC4682974; doi:10.1371/journal.pone.0144712)

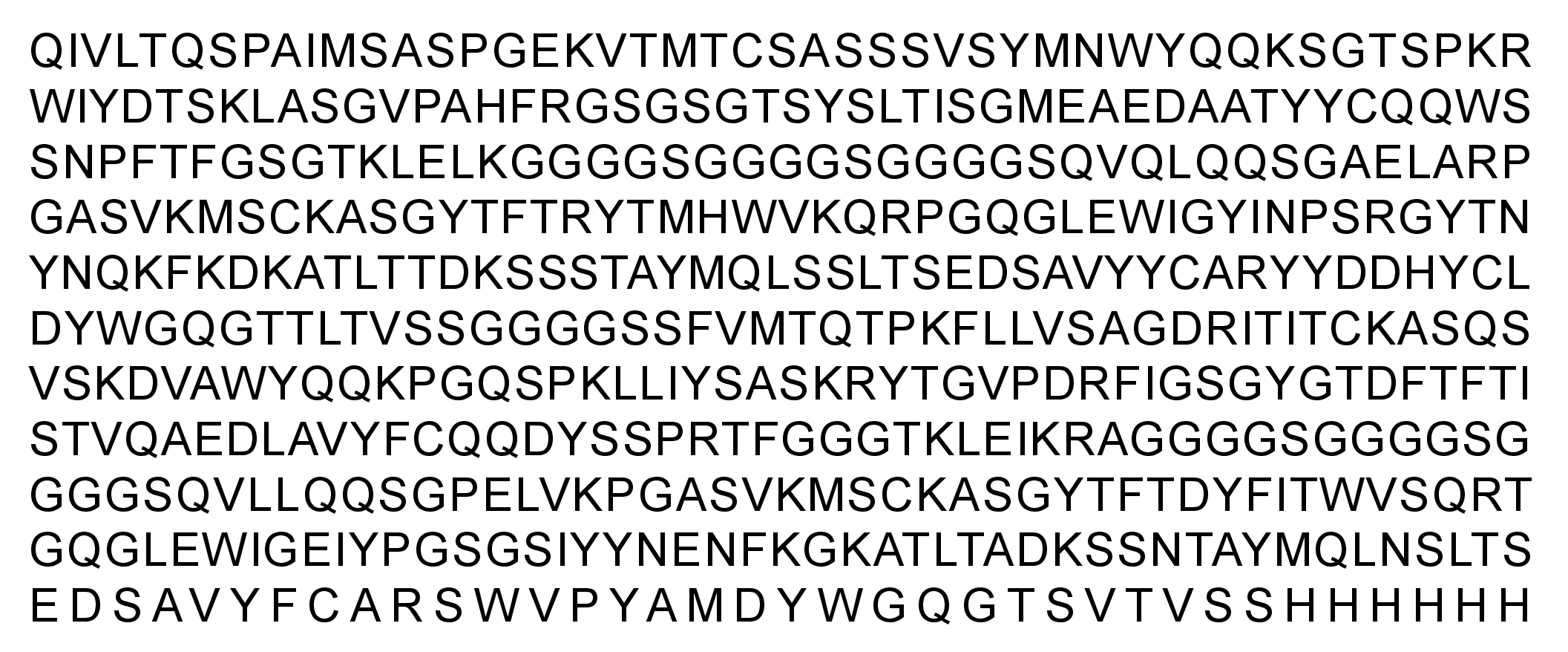

Supplement: S1 Fig — (TIF) [file pone.0144712.s001.tif]

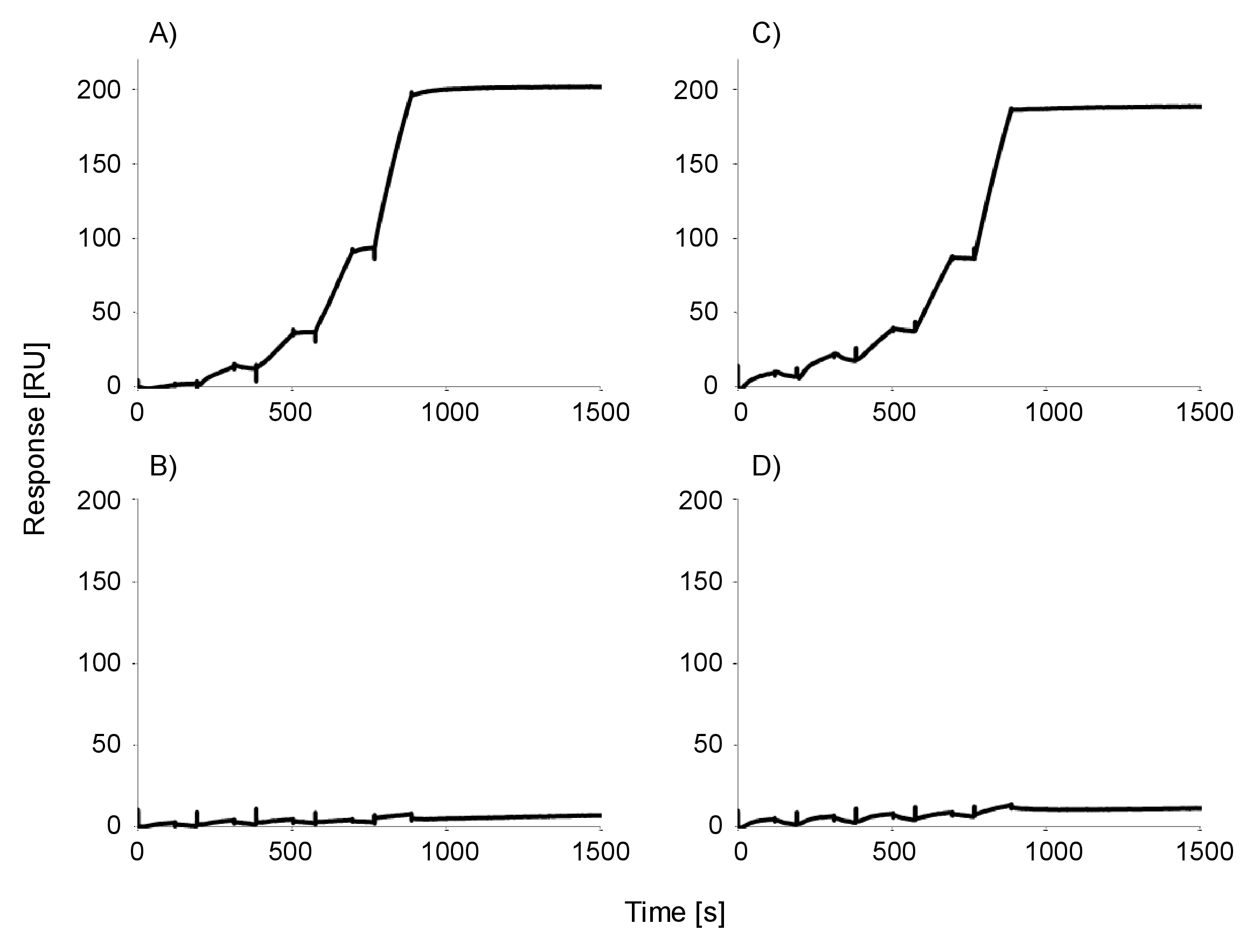

Supplement: S2 Fig — Binding curves were determined by surface plasmon resonance (SPR) measurements with a BiacoreT200 (GE Healthcare). Anti-human antibodies (Human Antibody Capture Kit; GE Healthcare) were immobilized on CM5 chips (at ~10,000 RU) using standard amine-coupling chemistry. After hEphA10-Fc (5 μg/mL) was captured on anti-human antibodies, each BsAb sample (1–240 nM) was injected into the flow cell. Binding response was corrected by subtracting RU from a blank flow cell. A) monomeric BsAb (EphA10/CD3), B) monomeric BsAb (His/CD3), C) dimeric BsAb (EphA10/CD3), D) dimeric BsAb (His/CD3). (TIF) [file pone.0144712.s002.tif]

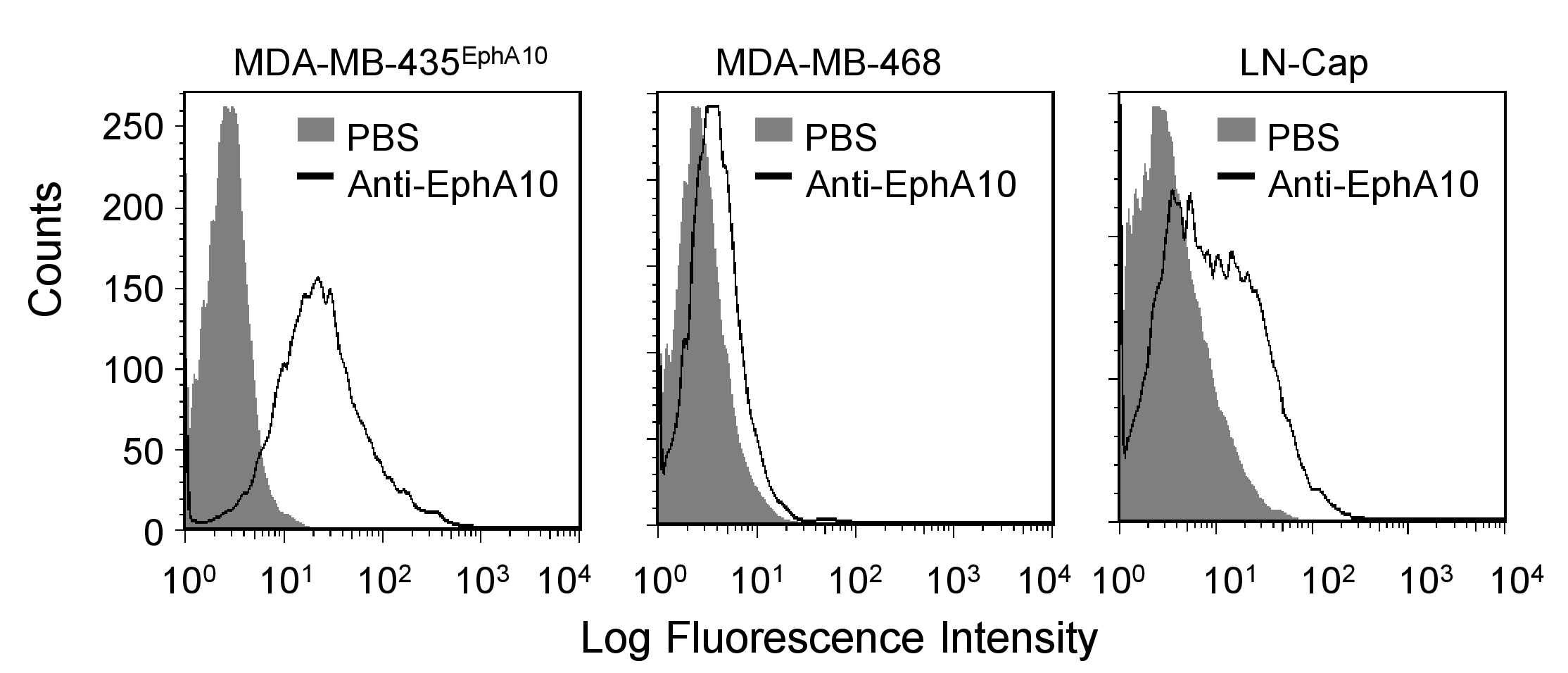

Supplement: S3 Fig — The method was describedd in the manuscript. (TIF) [file pone.0144712.s003.tif]

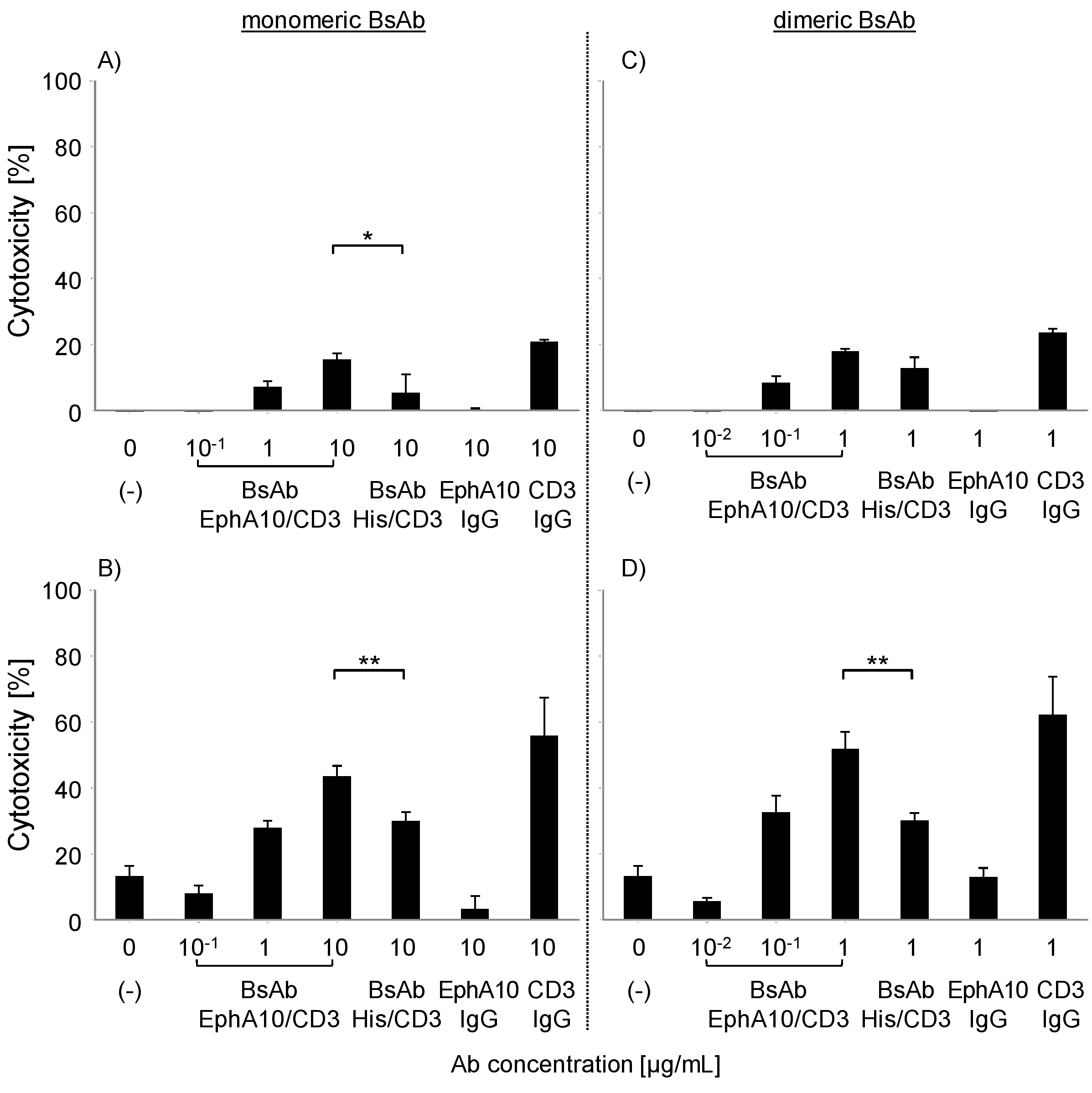

Supplement: S4 Fig — The left panels are monomeric BsAb (A, B) and the right panels are dimeric BsAb (C, D). Upper panels are MDA-MB-468 and lower panels are LN-Cap. Target cells were co-cultured with human PBMC at E/T ratios of 5. Each point represents the mean of triplicate determinations; Error bars represent the standard deviations of triplicate determinations. Asterisks label readings that were statistically significant (unpaired Student’s T-test) from BsAb (EphA10/CD3) and BsAb (His/CD3) (**: P<0.01, *: P<0.05). (TIF) [file pone.0144712.s004.tif]

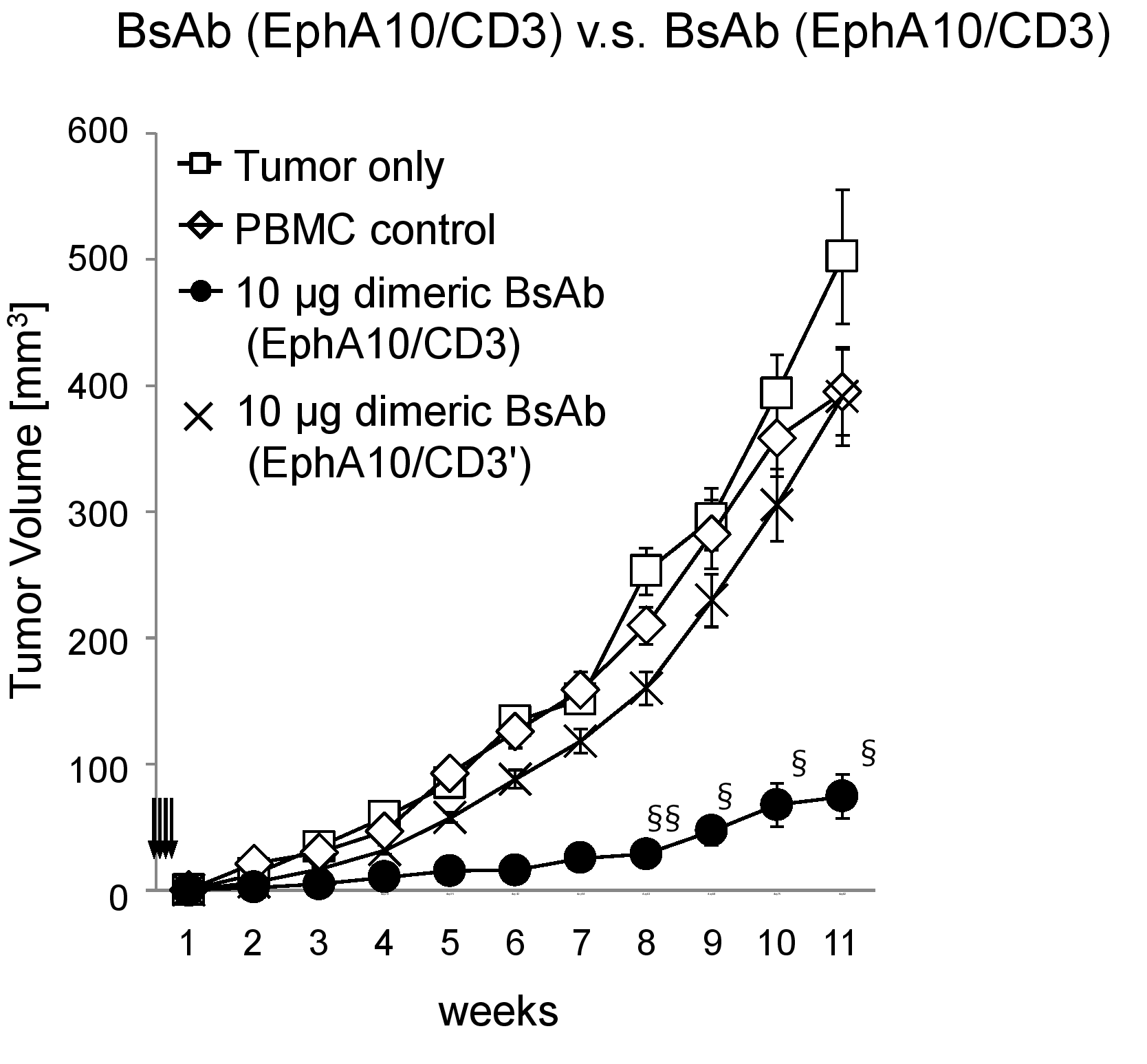

Supplement: S5 Fig — We evaluated the anti-tumor effect of another dimeric BsAb (EphA10/CD3’) that was constructed with anti-CD3 IgM. Each mouse (n = 6) was inoculated subcutaneously with a mixture of 106 MDA-MB-435EphA10 cells and 106 human PBMC at an E/T ratio of 1 and the indicated doses of dimeric BsAb were administered intravenously on study days 0 to 3 (arrows). Mean values of tumor growth curves are shown for mice that were untreated (⬜) or only PBMC-treated (◇), or treated with PBMC and 10 μg dimeric BsAb (EphA10/CD3) (●),10 μg dimeric BsAb (EphA10/CD3’) (×). Values represent mean tumor sizes (in mm3) ± SEM (n = 6 per group). Section signs indicate statistically significant differences from BsAb (EphA10/CD3) and BsAb (EphA10/CD3’) (§§: P<0.01, §: P<0.05). (TIF) [file pone.0144712.s005.tif]

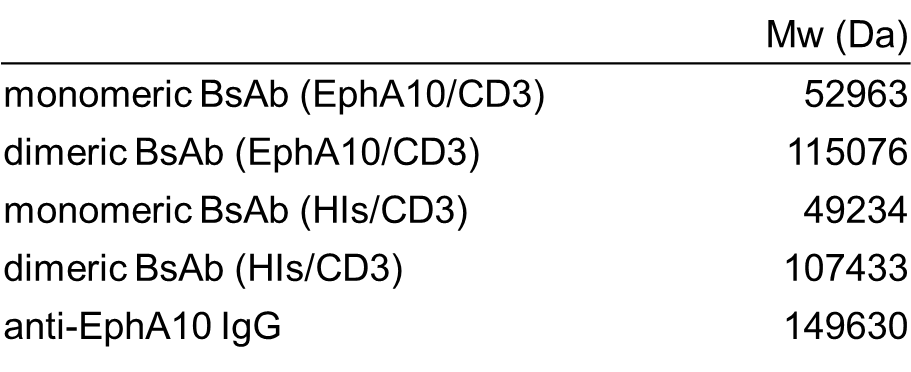

Supplement: S1 Table — Calibration curve was obtained by Gel Filtration Calibration Kit LMW and HMW (GE Healthcare). (TIF) [file pone.0144712.s006.tif]
